# A study of long-term potentiation in transgenic mice over-expressing mutant forms of both amyloid precursor protein and presenilin-1

**DOI:** 10.1186/1756-6606-3-21

**Published:** 2010-07-14

**Authors:** Stephen M Fitzjohn, Frederick Kuenzi, Robin A Morton, Thomas W Rosahl, Huw Lewis, David Smith, Guy R Seabrook, Graham L Collingridge

**Affiliations:** 1 MRC Centre for Synaptic Plasticity, Department of Anatomy, University of Bristol, University Walk, Bristol, BS8 1TD, UK; 2 The Neuroscience Research Centre, Merck Sharp and Dohme Research Laboratories, Terlings Park, Eastwick Road, Harlow, Essex, CM20 2QR, UK; 3 Merck Research Laboratories, 126 E. Lincoln Ave, Rahway, NJ 07065, USA; 4 Eli Lilly & Company, Lilly Corporate Center, Indianapolis, Indiana 46285, USA

## Abstract

Synaptic transmission and long-term potentiation (LTP) in the CA1 region of hippocampal slices have been studied during ageing of a double transgenic mouse strain relevant to early-onset familial Alzheimer's disease (AD). This strain, which over-expresses both the 695 amino acid isoform of human amyloid precursor protein (APP) with K670N and M671L mutations and presenilin 1 with the A246E mutation, has accelerated amyloidosis and plaque formation. There was a decrease in synaptic transmission in both wildtype and transgenic mice between 2 and 9 months of age. However, preparing slices from 14 month old animals in kynurenic acid (1 mM) counteracted this age-related deficit. Basal transmission and paired-pulse facilitation was similar between the two groups at all ages (2, 6, 9 and 14 months) tested. Similarly, at all ages LTP, induced either by theta burst stimulation or by multiple tetani, was normal. These data show that a prolonged, substantially elevated level of Aβ are not sufficient to cause deficits in the induction or expression of LTP in the CA1 hippocampal region.

## Background

Three loci have been identified that account for nearly all the familial Alzheimer's disease (AD) cases. Mutations in the amyloid precursor protein (APP) gene account for around 2-3% percent of familial AD cases and mutations in presenilins 1 and 2 (PS1 and PS2) have been linked to 70-80% of early onset AD [1-3]. The mutations associated with early onset familial AD in a Swedish family, where the 695 amino acid APP protein contains the two mutations K670N and M671L (APP_695_SWE mutation), affect cleavage of APP at the β-secretase site [4]. Subsequent cleavage of APP at the intramembranous γ-secretase site results in the formation of Aβ. PS1 is intimately associated with the cleavage of APP at the γ-secretase site and familial AD mutations in PS1, such as the A246E mutation occurring in transmembrane domain 6 of the presenilin protein [5,6], alter the efficiency of cleavage of APP and accelerate the production of Aβ [3,6-8].

Mice expressing the human form of the APP_695_SWE mutation develop certain AD-like symptoms, such as increased Aβ deposits and plaques, increased glial cell number, and deficits in spatial memory learning tasks [9-12]. Physiological studies have focused on the hippocampus, a region affected by AD, and have studied, in particular, long-term potentiation (LTP) as a candidate synaptic mechanism involved in learning and memory [13]. However, the findings have been controversial. Thus, one study reported normal synaptic transmission but impaired LTP in the APP_695_SWE mutant [12] whilst in other studies synaptic transmission was impaired but LTP was normal in the mutant [14,15]. In contrast, mice expressing the PS1_A246E _mutation showed enhanced LTP [16], along with increased production of Aβ(42), the longer form of Aβ associated with plaque formation [17].

Mice expressing mutations in both APP and PS1 exhibit accelerated Aβ production compared to those carrying mutations in only APP or PS1 [18]. Studies using double transgenic (DbTg) mice expressing both the APP_695_SWE mutations in APP and either the PS1_M146L _[19] or PS1_P264L _[20] mutation showed reduced hippocampal LTP at a younger age than basal synaptic transmission was compromised. However, a recent study found no alteration in hippocampal synaptic transmission or plasticity in mice overexpressing the APP_695_SWE and PS1_ΔE9 _mutations, even though the mice showed deficits in spatial learning [21]. Another DbTg strain of mouse which over-expresses both the 695 amino acid isoform of human amyloid precursor protein (APP) with K670N and M671L mutations (APP_695_SWE mice) and the A246E mutation in PS1 (PS1_A246E_) has much more aggressive amyloidosis and plaque deposition than the APP_695_SWE mice [17]. Given the relevance of these mouse models to human disease but the conflicting findings of the effect of double mutations on synaptic transmission and plasticity in the hippocampus, we report here an analysis of these DbTg animals.

## Methods

### Transgenic mice

The generation of the APP_695_SWE transgenic mice used in this study has been described previously [9,22]. APP_695_SWE transgenic mice were originally in a hybrid 87.5% C57BL6 x 12.5% SJL genetic background and were subsequently backcrossed to C57BL6 x SJL F1 mice over several generations. APP_695_SWE transgenic mice in a background closer to 50% C57BL6-50% SJL were then crossed with the PS1_A246E _transgenic line overexpressing the familial AD PS1_A246E _mutation of human PS1 [23] to generate the DbTg line (APP_695_SWE x PS1_A246E_; Lewis et al, 2004). All animals used in this study were from the N4 generation. All procedures were carried out in accordance with The UK Animals (Scientific Procedures) Act 1986.

Four age groups were studied: 2 months (i.e., between 2 and 3 months of age), 6 (6-7), 9 (9-10) and 14 (14 -15) months of age. Mice older than 15 months were not studied because of their high mortality and ethical considerations. All mice in this study were either heterozygous for both the APP_695_SWE and PS1_A246E _transgene (DbTg) or their wildtype (Wt) littermates. Animals were genotyped using PCR based methods for detection of the APP_695_SWE transgene [9,14,22], the PS1_A246E _transgene [23] and for the *rd *(retinal degeneration) mutation [24]. *Rd *homozygous mice were excluded from this study as a precaution, since it has been suggested that this mutation may indirectly affect neuronal number within the hippocampus [25]. All experiments and analyses were performed with the experimenters blind as to the genotype of the animal.

### Quantification of Aβ levels by HTRF

Levels of Aβ were determined by homogenous time-resolved fluorescence (HTRF) immunoassay as described previously [26]. Amyloid was extracted from the contralateral hemispheres by homogenisation in 10 volumes of 5 M GnHCl, 50 mM HEPES (pH 7.3), 5 mM EDTA plus 1x protease inhibitor cocktail (Complete™, obtained from Roche Diagnostics). Following 3 h rotation at room temperature, the homogenate was diluted ten-fold into ice-cold 25 mM HEPES (pH 7.3), 1 mM EDTA, 0.1% BSA plus 1x protease inhibitor cocktail and centrifuged (16,000g, 20 min, 4°C). Aliquots of the supernatant were stored at -20°C. The levels of amyloid peptides Aβ(40) and Aβ(42) were then detected by HTRF. All peptides (of > 95% purity; California Peptide Research Inc., California, U.S.A.) were frozen at 100 μM in 100% DMSO and serially diluted in buffer whose composition reflects that of the extracted samples (1 part GnHCl extraction buffer: 9 parts dilution buffer, as above). The HTRF signal was generated as a result of non-radiative transfer from europium cryptate-labelled Aβ (40)- or Aβ (42)-specific antibodies (G2-10 and G2-11 respectively; licensed from the University of Heidelberg; labelled at CIS bio international, Marcoule, France) to streptavidin-conjugated APC (Prozyme). The latter was brought into the complex by interaction with biotinylated antibody 4G8 (Senetek plc, Missouri, U.S.A.), which is specific for residues 17-24 of Aβ. Final reagent concentrations in a typical 96-well plate assay were: G2-10K (0.75 nM) or G2-11K (0.6 nM), 4G8 +/- biotin (1.0 nM), SA-XL665 (2.0 nM), KF (0.1-0.2 M). 50 μl of sample or synthetic peptide standard were assayed and a total volume of 200 μl/well was made up with dilution buffer. Blank values were determined by the use of non-biotinylated 4G8 antibody. The reaction mixture was left at 4°C for 20 h, and then read on the Discovery™ HTRF microplate analyser, providing simultaneous measurement at 665 nm (XL665 fluorescence) and 620 nm (EuK fluorescence). The ΔR ratio [= Ratio (sample) - Ratio (blank)] was used to extrapolate the amyloid concentrations of the brain extracts from the synthetic peptide standard curves.

### Histology

Histology to demonstrate plaque deposition was performed as described previously (Lewis et al, 2004). Briefly, 6 μm sagittal hippocampal slices were prepared and monoclonal anti-human β-amyloid (clone 6F3D) (DAKO, UK) was used to demonstrate amyloid plaques whilst polyclonal anti-GFAP (glial fibrillary acidic protein: DAKO) used to label reactive astrocytes. Endogenous peroxidase activity was blocked in 0.3% H_2_O_2 _in 0.1 M pH 7.4 phosphate buffered saline (PBS) for 30 min; non-specific binding was blocked by incubation with 5% normal horse serum (5% NHS) (Vector Labs, UK) in PBS for 1 h. Mouse anti-β-amyloid was applied (1:100 in 5% NHS) overnight at 4°C. Biotinylated anti-mouse IgG (Vector Labs) was applied for 30 min, followed by ABC reagent (Vector *Elite*, Vector Labs) for 30 min. DAB (Menarini, UK) was used as the chromogenic substrate. Peroxidase activity was quenched in 0.3% H_2_O_2_/PBS and sections further incubated in polyclonal anti-GFAP (1:1000 in 5% normal goat serum) overnight at 4°C. Visualisation was achieved by incubation in biotinylated anti-rabbit IgG followed by ABC reagent (Vector *Elite*, Vector Labs).

### Electrophysiology

Recordings were made form 350 μm thick hippocampal slices prepared from 2, 6, 9 and 14 month old DbTg and Wt mice. Animals were killed by decapitation, as licensed under the UK Animals (Scientific Procedures) Act 1986, and the brains rapidly removed in ice-cold artificial cerebrospinal fluid (aCSF). The composition of this aCSF was, in mM: NaCl, 126; NaH_2_PO_4_, 1.2; MgCl_2_, 1.3; CaCl_2_, 2.4; KCl, 2.5; NaHCO_3_, 26; glucose, 10. Brains were cut along the midline and parasagittal whole brain slices prepared from one hemisphere (randomly chosen) using a Vibratome. The hippocampus was then dissected out of these slices. The contralateral hemisphere was used for histology or determination of Aβ levels. Slices were allowed to recover for at least 1 h at room temperature before being transferred to a submerged recording chamber perfused with aCSF at 2 ml.min.^-1 ^and maintained at 33°C. Kynurenic acid (1 mM) was included in the aCSF used for preparing slices from the 14 month old animals. Slices were then transferred to non-kynurenic acid containing aCSF approximately 30 min after dissection and allowed to recover for a further 30 min.

Field excitatory postsynaptic potentials (fEPSPs) were recorded from stratum radiatum of area CA1 and Schaffer collateral-commissural fibres were stimulated using a bipolar nickel-chromium electrode. The initial slope of the negative-going phase of the fEPSP was used as a measure of synaptic efficacy. Recordings were made using a SPIKE 2 script running on a CED1401plus interface (Cambridge Electronic Design). Stimulus-response curves were constructed by using stimulus intensities from 0 to 45 V in increments of 5 V. Responses were subsequently set to a level that gave a slope value of approximately 20% of the maximum obtained. Baseline responses were obtained every 30 s. Paired-pulse facilitation (PPF) was assessed using a succession of paired-pulses using inter-pulse intervals of 25, 50, 100, 200 and 300 ms. A further 30 min baseline period was obtained before attempting to induce LTP.

LTP was induced by delivery of a theta burst stimulation paradigm (TBS), which comprised 10 bursts, at an interburst frequency of 5 Hz and an intraburst frequency of 100 Hz, each burst consisting of 4 stimuli delivered at test stimulus intensity. In some experiments, LTP was induced using a stronger stimulation whereby a repetitive tetanic stimulus was applied (100 stimuli at 100 Hz at test intensity repeated 4 times at 5 min intervals). All data are presented as mean ± s.e.mean. n values are given as [x (y)] where x = number of slices and y = number of animals. Data were log transformed and analysed using a one-way ANOVA with repeated measures (BMDP statistical package, release 7). Linear regression of fEPSP slope vs fibre volley amplitude was done with Microsoft^® ^Excel 97 Analysis Toolpack.

## Results

### Aβ levels and amyloid plaque deposition

**Figure 1:**
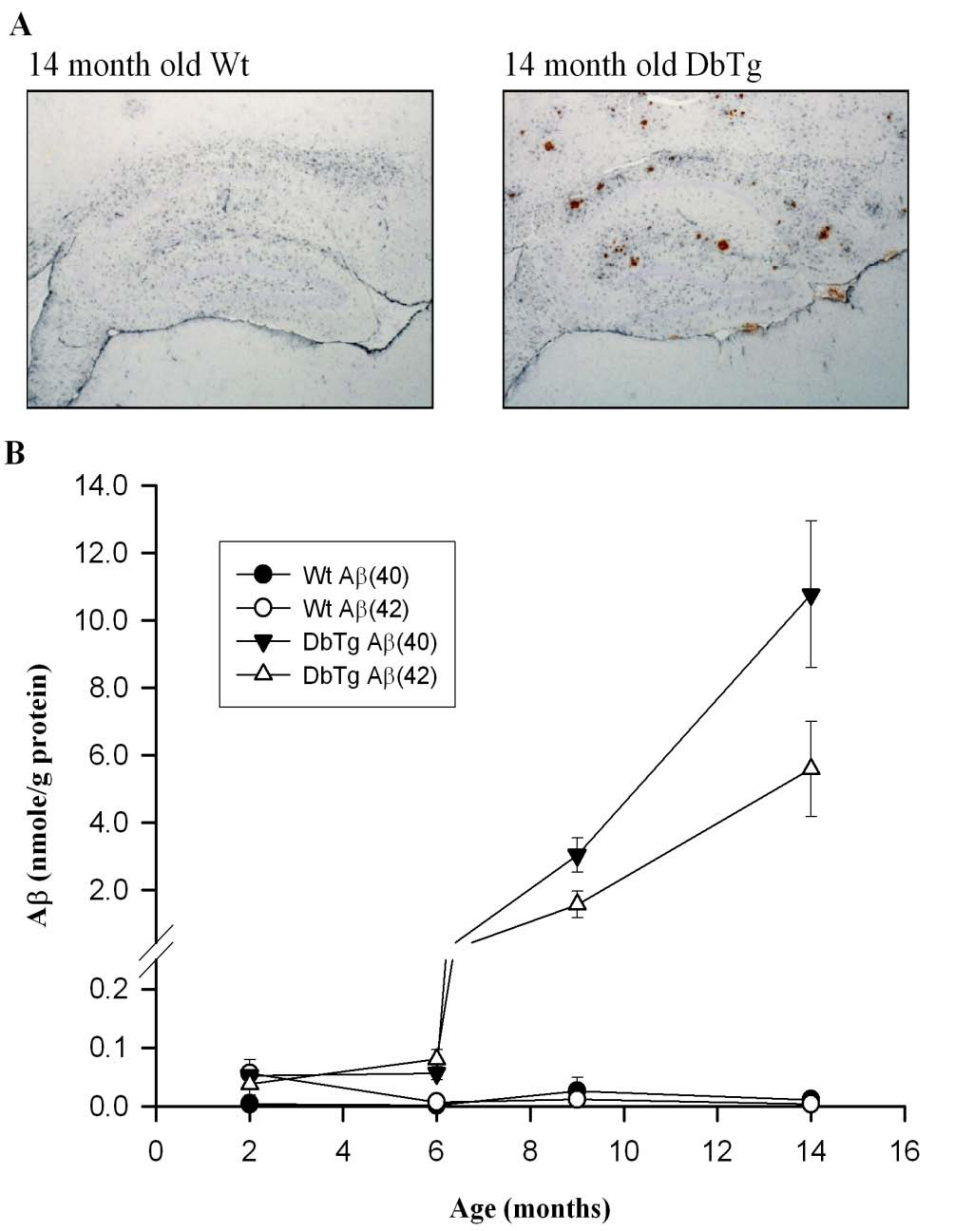
**Plaques and elevated levels of Aβ(40) and Aβ(42) in DbTg (Tg) mice**. (A) Example hippocampal slices from 14 month old animals, illustrating plaque formation in the Dbtg mice (brown) accompanied by astrocytosis (black). (B) Pooled data showing levels of Aβ(40) and Aβ(42) in Wt and DbTg mice.

Expression of the APP_695_SWE x PS1_A246E _transgenes produced a dramatic increase in both short (Aβ(40)) and long (Aβ(42)) forms of the Aβ peptide (Fig. 1). This elevation in Aβ levels occurred several months earlier than in the single APP_695_SWE transgenic [14,26]. At 9 months, the Aβ load was significantly greater than in Wt brains; levels of Aβ(40) were 3.0 ± 0.5 compared with 0.03 ± 0.02 nmol/g wet weight tissue and levels of Aβ(42) were 1.6 ± 0.4 compared to 0.01 ± 0.009 nmol/g wet weight tissue, in DbTg and Wt mice, respectively. At 14 months the levels of Aβ(40) and Aβ(42) increased to 10.8 ± 2.2 and 5.6 ± 1.4 nmol/g wet weight tissue in DbTg mice, compared to 0.01 ± 0.01 and 0.004 ± 0.003 nmol/g wet weight tissue in Wt mice (Fig. 1B). In addition, compared to the single transgenic APP_695_SWE mouse, the Aβ(42)/Aβ(40) ratio is enhanced throughout the life of the animal [14,26]. Plaques were also evident in aged DbTg mice throughout the hippocampus, but were not evident in Wt animals (Fig. 1A; [26]).

### Basal synaptic transmission

**Figure 2:**
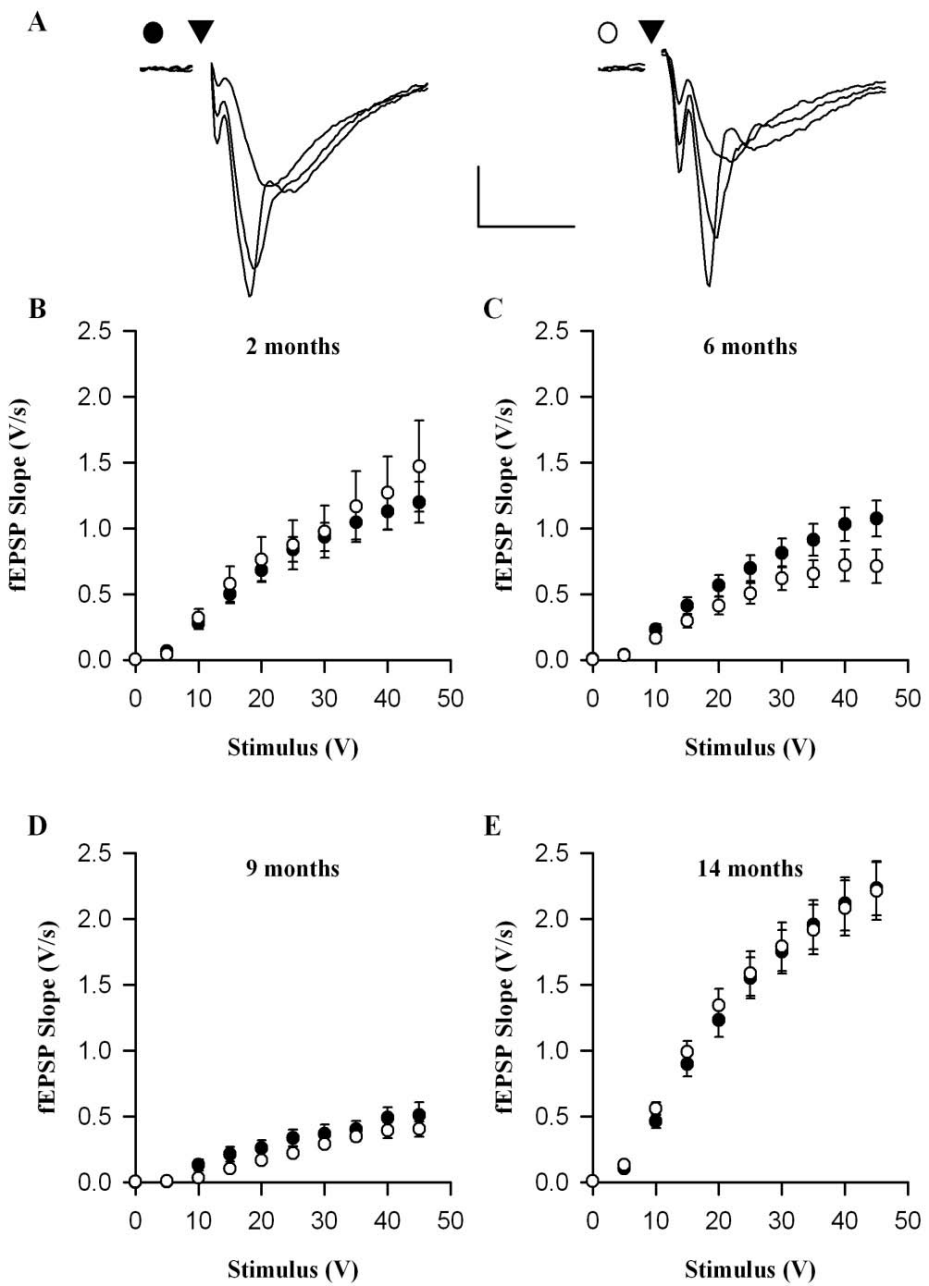
**Age-dependent reduction in synaptic transmission in CA1 region of Wt and DbTg mice**. (A) Individual examples of fEPSPs recorded with a stimulus intensity of 10, 20 and 30 V from slices prepared from 14 month old Wt and DbTg mice. Scale bar 0.5 mV, 10 ms. Stimuli were delivered at the times indicated by the arrows and stimulus artefacts have been removed for clarity. (B - D) Pooled data showing relationship between fEPSP slope and stimulus intensity at 2, 6, 9 and 14 months. In this and following figures symbols are [black circle] for Wt and [white circle] for DbTg results.

**Figure 3:**
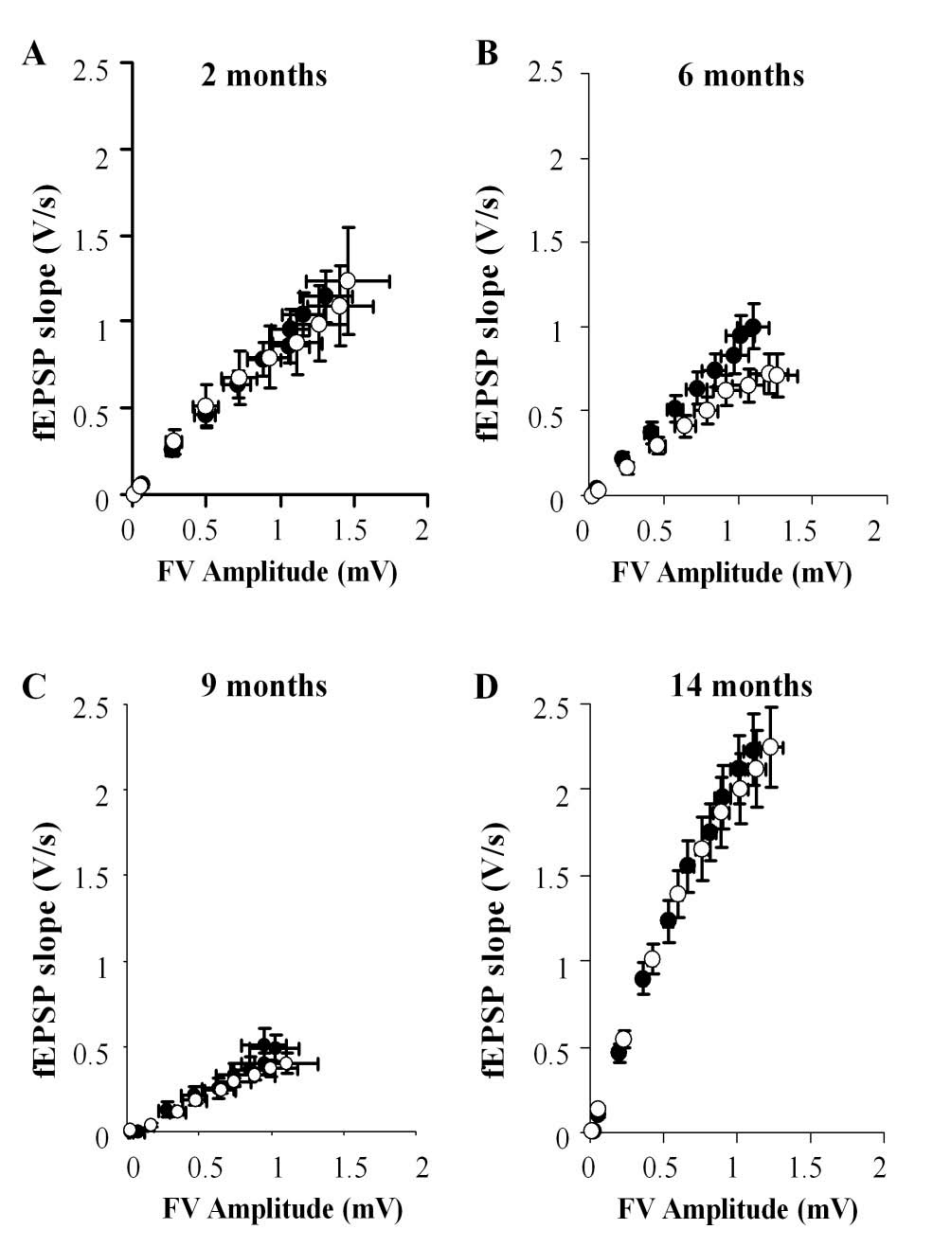
**Analysis of fibre volley (FV) amplitude and fEPSP slope**. Pooled data from 2, 6, 9 and 14 month old Wt and DbTg animals respectively grouped by stimulus intensity ([black circle] Wt, [white circle] DbTg).

Synaptic transmission was quantified over a wide range of stimulus intensities and was analysed in terms of fEPSP slope *versus *stimulus intensity (Fig. 2) and fEPSP slope *versus *fibre volley (FV) amplitude (Fig. 3). Using both methods of analysis there was a pronounced, age-dependent decline in synaptic transmission in slices obtained from both Wt and DbTg mice of between 2 and 9 months of age. Indeed, it was difficult to obtain viable synaptic responses from slices prepared form both Wt and DbTg animals in the 9 month age group and many failed to produce a fEPSP. For this reason we performed the dissections for the 14 month age group in the presence of kynurenic acid, since this treatment has been shown to greatly improve slice viability of some transgenic mouse strains [12,14]. This treatment resulted in considerably improved synaptic viability (Figs 2 &3).

The input-output curves were generally similar for both genotypes, at all ages studied (Figs 2 &3). For example, in 14 month old mice the slope of the relationship between fEPSP slope and FV amplitude was 2.34 ± 0.25 V.s^-1^.mV^-1 ^[47 (11)] in Wt animals and 2.42 ± 0.31 V.s^-1^.mV^-1 ^[43 (13)] in DbTg mice (p > 0.05). The maximum obtained fEPSPs were also similar in the two groups. For example, in 14 month old mice the fEPSP evoked by 45 V stimulation was 2.32 ± 0.21 V.s^-1 ^[47 (11)] and 2.21 ± 0.22 V.s^-1 ^[43 (13)] (p > 0.05) in Wt and DbTg mice, respectively.

### Paired-pulse facilitation

**Figure 4:**
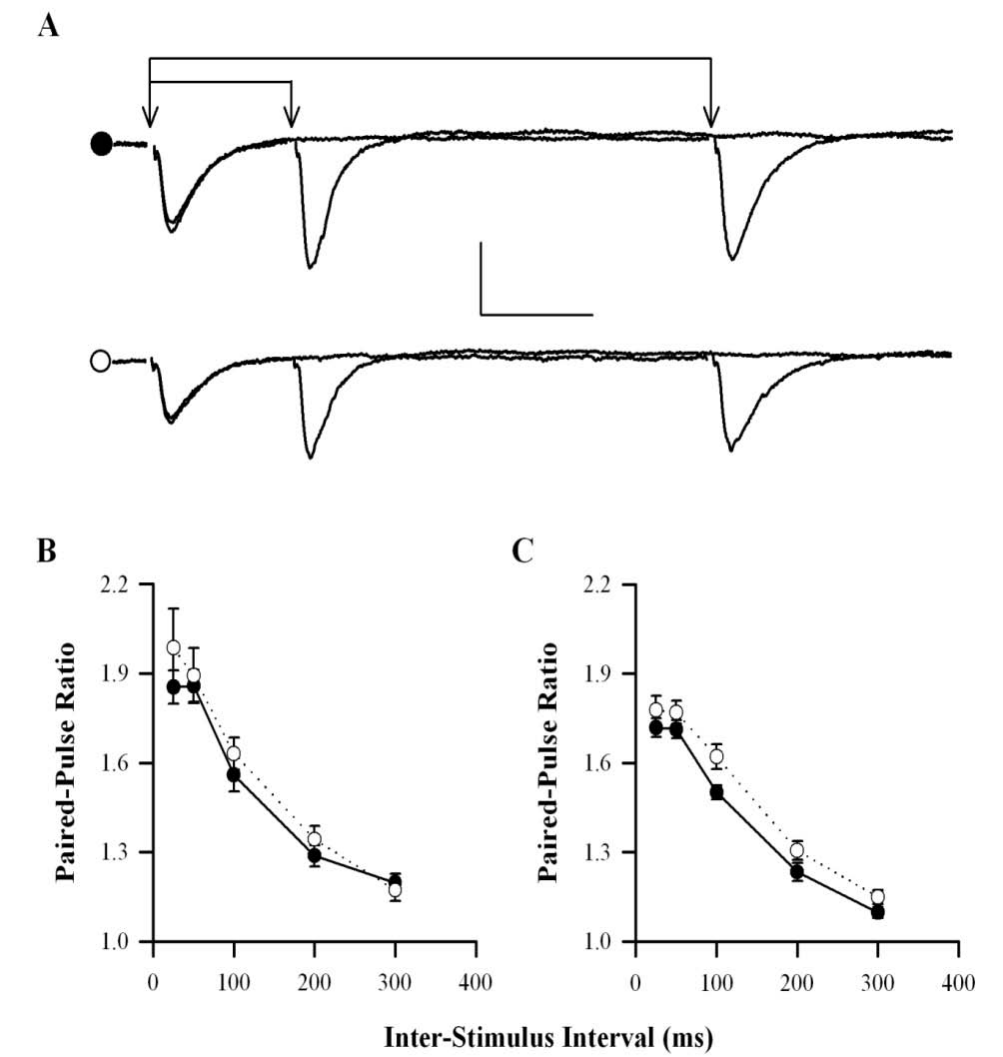
**PPF is normal in DbTg mice**. (A) Example traces taken from 14 month old Wt (black circle) and DbTg (white circle) animals showing PPF at ISIs of 25 and 100 ms. Scale bar 0.5 mV, 20 ms. (B, C) Pooled data for 2 (B) and 14 (C) month-old animals.

In all age groups, there was no difference in the level of PPF (slope of second response/slope of first response) between DbTg and Wt mice. For example, at an inter-stimulus interval (ISI) of 50 ms the PPF ratios for Wt and DbTg mice were, respectively, at 2 months: 1.86 ± 0.05 [30 (13)] and 1.99 ± 0.13 [20 (10)]; 6 months: 1.93 ± 0.07 [24 (8)] and 1.80 ± 0.15 [15 (7)]; 9 months: 1.81 ± 0.10 [28 (10)] and 1.75 ± 0.09 [19 (8)] and 14 months: 1.72 ± 0.03 [28 (8)] and 1.77 ± 0.04 [24 (6)] (in all cases, p > 0.05). There was also no age-related change in the level of PPF in either Wt or DbTg animals (p > 0.05 at all stimulus intervals used). PPF data over a range of inter-pulse intervals for the 2 and 14 month age group are presented in Fig. 4.

### Long-term potentiation

**Figure 5:**
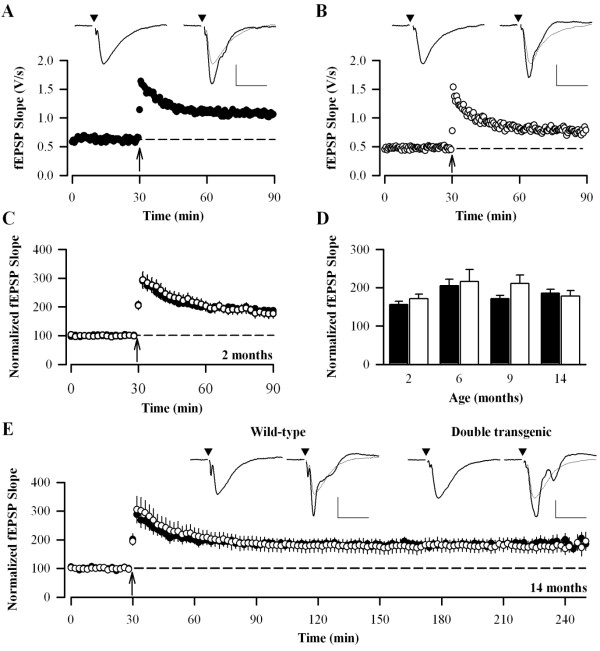
**LTP induced by a theta burst stimulus is normal in DbTg mice**. (A) Example experiment taken from 14 month old Wt slice. LTP was induced at the time indicated by the arrowhead. Example traces are taken from the time points immediately prior to and 60 min after the induction of LTP. Scale bar 0.5 mV, 20 ms. (B) Example experiment taken from 14 month old DbTg slice. (C) Pooled data for 2 month-old animals ([black circle] Wt, [white circle] DbTg). In this and subsequent figures showing pooled data, data points are shown at two minute intervals. (D) Summary of LTP as a percentage of baseline at 60 min post-tetanus for the different age groups. (E) Pooled data from a subset of slices from 14 month old animals where LTP was followed for 3.5 h after the theta burst. Scale bar 0.5 mV, 10 ms.

A theta burst stimulus (total of 40 stimuli) induced robust LTP in the CA1 region in all age groups. The level of LTP was similar in both genotypes across all ages, quantified for up to 60 min following theta burst stimulation (Fig. 5A-D; p > 0.05 at all age groups). For example, the level of LTP observed 60 minutes after theta burst stimulation in Wt and DbTg animals was, at 2 months: 156 ± 9% [9 (5)] and 171 ± 12% [8 (6)]; 6 months: 205 ± 17% [18 (9)] and 216 ± 32% [13 (7)]; 9 months: 171 ± 9 [15 (8)] and 211 ± 22% [8 (4)]; 14 months: 186 ± 10% [23 (13)] and 178 ± 15% [16 (9)]. There was no age-related change in the level of LTP seen following theta burst stimulation for either Wt of DbTg mice. In the 14 month age group, a subset of slices was followed for over 3 h following theta burst stimulation. There was no difference between genotypes, the level of LTP seen 3.5 hours post-theta burst stimulation in Wt and DbTg animals was 196 ± 28% [9 (5)] and 183 ± 20% [9 (5)] (p > 0.05; Fig. 5E).

**Figure 6:**
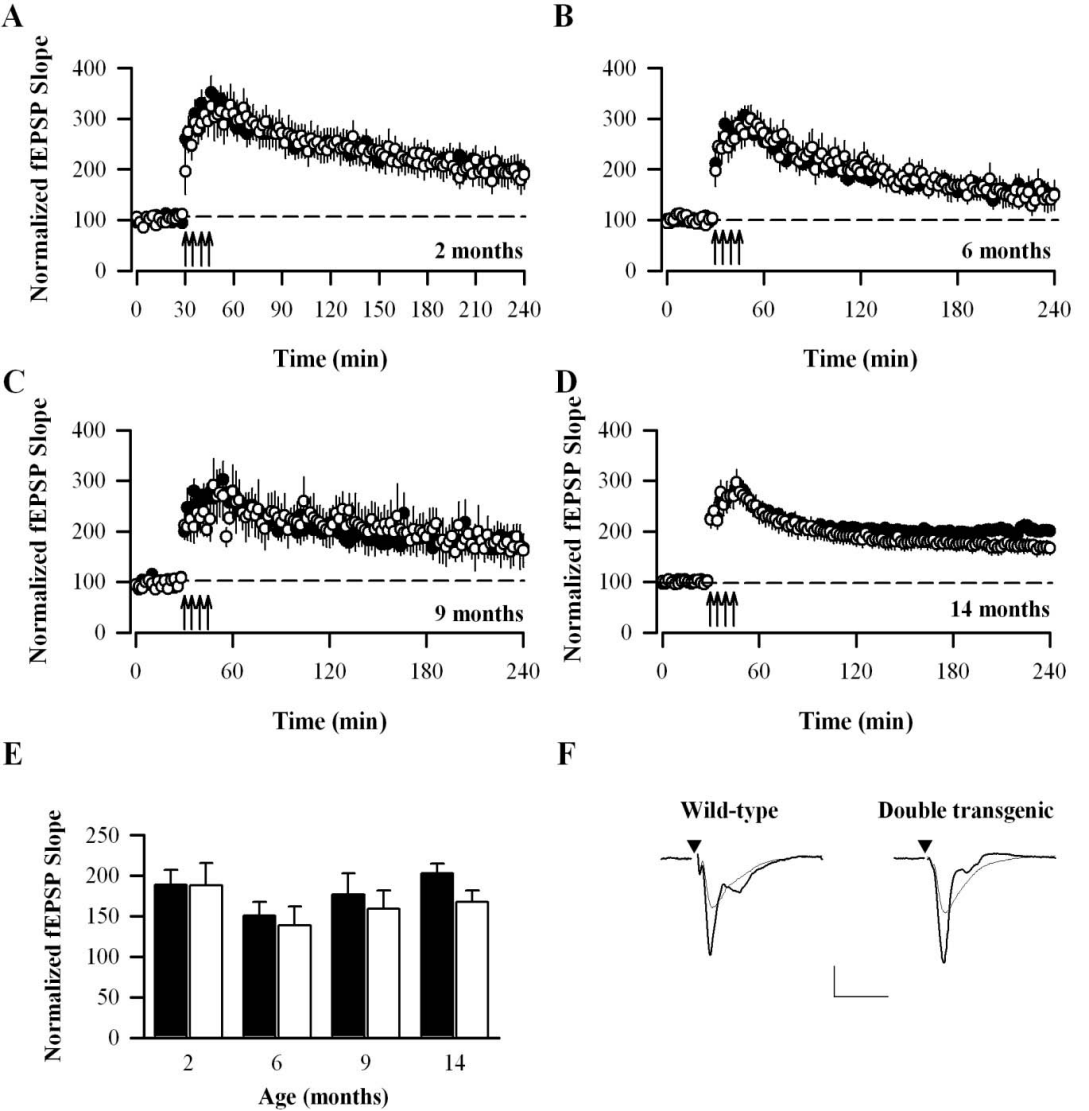
**LTP induced by repeated tetani is normal in DbTg mice**. (A-C) Pooled data for slices from 2, 6 and 9 month old animals ([black circle] Wt, [white circle] DbTg). (D) Summary of the LTP as a percentage of baseline at 3.5 hours after the last tetanus for the different age groups. (E) Pooled data for slices from 14 month old animals. Traces are taken from the time points immediately prior to induction of LTP and 3.5 h after the last tetanus. Scale bar 0.5 mV, 5 ms. LTP was induced by the delivery of four tetanic stimuli separated by 5 min at the times indicated by the arrowheads.

In a second series of experiments, a stronger induction protocol was delivered (total of 400 stimuli) and LTP again followed for over 3 h (Fig. 6). The levels of LTP were similar for each genotype at all ages (p > 0.05 at 3.5 h), thus at 3.5 hours post-LTP induction the level of LTP in Wt and DbTg animals was, at 2 months: 189 ± 18% [10 (5)] and 188 ± 28 [7 (3)]; 6 months 151 ± 17% [11 (9)] and 139 ± 23% [8 (5)]; 9 months: 177 ± 26% [11 (6)] and 159 ± 23% [9 (5)]; 14 months: 203 ± 12% [20 (9)] and 168 ± 14% [16 (8)]. Again, there was no age-related change in the level of LTP induced by this strong induction protocol for either Wt or DbTg animals.

## Discussion

In DbTg mice over-expressing the human familial AD transgenes APP_695_SWE and PS1_A246E_, Aβ peptides accumulate in an accelerated age-dependent manner with an early enhancement of the Aβ(42)/Aβ(40) ratio (see also [26]). Accelerated production of Aβ has also been reported in different mouse strains expressing double mutations of APP and PS1 (reviewed in [18]). However, there has been little work carried out into studying synaptic function and plasticity in these multiple transgenic mice lines. We find that normal LTP in the CA1 region can be recorded over a wide age range and that there is little, if any, difference between the DbTg and the Wt mice.

### Effects on basal synaptic transmission

Consistent with our earlier report on the APP_695_SWE single transgenic mouse [14] we found that there was an age-dependent reduction in fEPSPs evoked by a fixed stimulus intensity over a wide range. The deficit was clearly present when synaptic transmission was compared with the amplitude of the presynaptic fibre volley and therefore reflects a reduction in the input-output relationship. This age-related decrease in basal synaptic transmission was similar in both Wt and DbTg mice, with little if any difference between the two groups at each age point. Similarly, Volianskis et al. [21] recently reported a similar age-related decrease in basal transmission in mice overexpressing APP_695_SWE and PS1_ΔE9 _transgenes, which was similar in both groups of animals. Consistent with previous studies [12,14], the reduction in synaptic transmission was prevented by blockade of ionotropic glutamate receptors with kynurenic acid during slice preparation, suggesting that it was due to increased susceptibility to excitotoxicity, which may be a characteristic of the background mouse strain on which the transgenes are expressed. Interestingly, hippocampal cultures prepared from double transgenic animals (APP_SWE _and PS1_L166P_) show a reduction in excitatory synapses compared to cultures from wildtype or APP_SWE _mice, suggesting that the inclusion of the PS1 transgene may have an additional effect on glutamatergic synapse formation under some conditions [27].

Here we have shown that, even at 14 month of age, there is no deficit in basal transmission in DbTg compared to Wt animals. This is despite the fact that Aβ levels are greatly enhanced in these animals and plaques are also evident in the hippocampus and other brain regions (see [26]). Thus it appears that the increased production of Aβ in these mice does not impair basal synaptic function.

### Effects on LTP

Previous studies using transgenic mouse modes of Alzheimer's disease have resulted in little consensus on the effects of mutations in APP and PS1 with respect to LTP. For example, a reduction of LTP has been reported in mice that over-express either the London (V642I; [28] or Swedish (APP_695_SWE; [12,29] mutations in APP. In contrast, no impairment was observed in single transgenic mice that over-express the V717F mutant form of APP (APP_Ind_; [30-32]) and in studies using the APP_695_SWE mutation [14,15]. Both the ΔE9 [33] and A246E [16] mutations in PS1 have previously been shown to lead to enhanced LTP expression. Mice under-expressing PS1 have displayed reduced levels of LTP in one study [34] but normal LTP in another [35].

Few studies to date have been conducted using mice expressing double mutations in APP and PS1. One previous study reported an increased rate of decay in dentate gyrus LTP *in vivo *in 17-18 month old mice expressing the APP_695_SWE and PS1_A246E_, mutations [36], but normal CA1 LTP *in vitro *at this age. Two studies have found impairments in hippocampal LTP [37] and hippocampal-dependent learning [37,38] in both aged wildtype and transgenic mice overexpressing mutated APP and PS1, suggesting that deficits were independent of Aβ or plaque load. Although impairments in CA1 LTP have been observed in mice expressing mutations in both APP and PS1 in some studies [19], [39], a recent study using mice expressing both APP_695_SWE and PS1_ΔE9 _found no deficit in hippocampal LTP *in vitro *at all ages studied (up to 12 months of age;[21]). In our study we were unable to detect any changes in LTP at CA1 synapses using two induction protocols (that employed respectively 40 and 400 stimuli). Of course, we cannot exclude the possibility that alterations in LTP may be observed with other patterns of activation or under different experimental conditions or in other pathways. Interestingly, a recent study has shown that deficits in LTP *in vitro *in mice overexpressing the Swedish mutant of APP are only seen if the animals are previously exposed to spatial training, not in naïve animals [40], suggesting that deficits in plasticity are subtle and subject to alterations based on prior experience. The primary conclusion, however, is that LTP is readily induced despite the pronounced and long-lasting increase in Aβ(42) and Aβ(40) levels in the hippocampus.

Alzheimer's disease is characterised by synaptic degeneration and changes in dendritic and axonal morphology [41-43]. Whilst these processes are not important for the earliest phase of LTP, they may become more important in the protein synthesis dependent phase of LTP. For this reason we extended our analysis of LTP beyond that which has been studied previously in slices obtained from transgenic models of Alzheimer's disease. However, we noticed little difference in LTP between the genotypes, even when followed for over 3 h post-induction. Theta patterns of activity are more physiological than tetanic stimulation and involve the activation of presynaptic GABA_B _receptors to transiently suppress GABA inhibition and thereby facilitate the activation of NMDA receptors [44]. Thus, LTP induced by theta patterns of activity may be more susceptible to regulatory influences, in particular those that affect GABA-mediated inhibition. In this context, we have previously observed that a deficit in LTP observed in APP null nice is normalised when GABA_A _receptor-mediated inhibition is blocked [45].

The finding that both basal synaptic transmission and LTP is normal in region CA1 of the hippocampus even when Aβ levels are greatly enhanced and plaques are present may be considered somewhat surprising, particularly as the presence of plaques would be expected to disrupt the neuronal organisation of this region. However, these findings are consistent with some of the previous studies utilising transgenic mice relevant to AD. This would suggest that synaptic physiology remains normal in situations where Aβ levels are elevated, at least in the CA1 region of the hippocampus. However, our experiments were performed without visualisation of plaques in the slices and thus we cannot rule out the possibility that the majority of our recording sites were in areas not containing plaques and that recording from regions neighbouring plaques may reveal a deficit in basal synaptic transmission and/or plasticity. This may also account for discrepancies between studies of transgenic mice expressing AD related proteins. Thus further experiments could target recordings from plaque containing regions. Ours and other studies investigating synaptic physiology from acutely prepared brain slices give an indication of transmission at individual or sets of synapses but do not provide information on the function of the hippocampus as a whole. If transmission and/or plasticity are impaired at only a fraction of the neurones in this structure then function (e.g. spatial learning) may be impaired.

Increasing evidence suggests that the form in which Aβ is present in the brain is crucial in terms of whether neuronal function and memory is impaired. Thus oligomers rather than monomers or fibrils of Aβ induce synapse loss and impair memory [46-48]. Aβ exists in the AD brain as a polydisperse mixture of high order oligomers and only these high molecular weight oligomers bind to hippocampal neurons [49]. Furthermore, cerebral injection of cell medium containing oligomers and monomers, but not fibrils, inhibits LTP *in vivo *[46]. As such the existence of Aβ oligomers rather then plaques may cause neuronal damage, and there is little correlation between the presence of plaques and severity of dementia in humans [50]. It may be, therefore, that although the mice used in the current study display Aβ plaques, they do not produce an oligomeric form of Aβ that is detrimental to neuronal health and function. Alternatively, it may be that a loss of normal APP function rather than an increase in Aβ may cause an impairment in LTP, as APP knockout mice show deficits in LTP [45], and so differences in secreted APP between transgenic strains, which can have trophic and neuroprotective effects [51], may lead to differences in synaptic transmission and plasticity.

Another possibility for our finding, a lack of effect on LTP in the DbTg mice, is that other factors may be required for generating AD-related impairments in hippocampal function, for example altered tau [52]. Consistent with this Roberson and colleagues have shown that reducing tau expression can affect amyloid related toxicity in transgenic mice without altering plaque deposition [53]. Human patients have additional pathology relative to transgenic mouse models utilising mutations in APP and PS1. In particular, although the mice show Aβ plaques, they do not display intracellular tangles of hyperphosphorylated tau protein [54]. Oddo and colleagues [55] generated a triple transgenic mouse expressing mutated APP (APP_SWE_), PS1 (M146V mutation) and tau (P301L mutation), which showed progressive development of both plaques and tangles as well as a reduction in hippocampal LTP. Such mice may represent a more robust model of AD.

## Conclusions

In conclusion, it is clear from our present experiments that the cellular machinery for induction and expression of LTP at CA1 synapses is intact in these DbTg mice. This is apparent even in aged mice, which have been exposed to greatly elevated levels of Aβ peptides for over 6 months.

## Competing interests

Frederick Kuenzi, Thomas Rosahl, David Smith, Huw Lewis and Guy Seabrook were employees of Merck Research Laboratories when this work was carried out. There are no other conflicts of interest.

## Authors' contributions

Conceived and designed the experiments: SMF, FK, RAM, TWR, GRS, GLC

Performed the experiments: FK, RAM, HL, DS, GRS

Analyzed the data: SMF, FK, RAM, GRS

Wrote the paper: SMF, FK, RAM, GRS, GLC.
